# A reassortant H9N2 influenza virus containing 2009 pandemic H1N1 internal-protein genes acquired enhanced pig-to-pig transmission after serial passages in swine

**DOI:** 10.1038/s41598-017-01512-x

**Published:** 2017-05-02

**Authors:** José Carlos Mancera Gracia, Silvie Van den Hoecke, Juergen A. Richt, Wenjun Ma, Xavier Saelens, Kristien Van Reeth

**Affiliations:** 1 Laboratory of Virology, Department of Virology, Parasitology and Immunology, Faculty of Veterinary Medicine, Ghent University, Merelbeke, Belgium; 2 Center for Medical Biotechnology, VIB, Ghent Belgium; 3 Department of Biomedical Molecular Biology, Ghent University, Ghent, 9000 Belgium; 4 Department of Diagnostic Medicine/Pathobiology, College of Veterinary Medicine, Kansas State University, Manhattan, KS 66506 USA

## Abstract

Avian H9N2 and 2009 pandemic H1N1 (pH1N1) influenza viruses can infect pigs and humans, raising the concern that H9N2:pH1N1 reassortant viruses could emerge. Such reassortants demonstrated increased replication and transmissibility in pig, but were still inefficient when compared to pH1N1. Here, we evaluated if a reassortant virus containing the hemagglutinin and neuraminidase of A/quail/Hong Kong/G1/1997 (H9N2) in the A/California/04/2009 (pH1N1) backbone could become better adapted to pigs by serial passaging. The tropism of the original H9N2:pH1N1 (P0) virus was restricted to the nasal mucosa, with no virus detected in the trachea or lungs. Nevertheless, after seven passages the H9N2:pH1N1 (P7) virus replicated in the entire respiratory tract. We also compared the transmissibility of H9N2:pH1N1 (P0), H9N2:pH1N1 (P7) and pH1N1. While only 2/6 direct-contact pigs showed nasal virus excretion of H9N2:pH1N1 (P0) ≥five days, 4/6 direct-contact animals shed the H9N2:pH1N1 (P7). Interestingly, those four animals shed virus with titers similar to those of the pH1N1, which readily transmitted to all six contact animals. The broader tissue tropism and the increased post-transmission replication after seven passages were associated with the HA-D225G substitution. Our data demonstrate that the pH1N1 internal-protein genes together with the serial passages favour H9N2 virus adaptation to pigs.

## Introduction

H9N2 avian influenza viruses are currently endemic in poultry in South East Asia and the Middle East^1^. Since the late 1990s, H9N2 viruses have been occasionally isolated from humans and swine in China, leading to increasing concerns about their pandemic potential^2–8^. However, all reported human or swine H9N2 virus infections were dead-end events and the virus failed to spread within the human or swine population^9^.

A significant proportion of poultry H9N2 field isolates contains a leucine instead of glutamine residue at position 226 in the hemagglutinin (HA) receptor-binding site (RBS) (HA-Q226L, H3 numbering used throughout). This mutation has been shown to enhance HA binding to terminal *α*2,6-linked sialic acids (Sia*α*2,6Gal) and to improve the replication of H9N2 viruses in human airway epithelial cells *in vitro*, supporting concerns about their pandemic potential^10–12^. The binding of the viral HA to sialyloligosaccharides, present on the host cell surface, is the first step for influenza virus infection. Because of the predominant expression of Sia*α*2,6Gal in human and swine upper respiratory tract^13, 14^, most human and swine influenza viruses more willingly bind to receptors that contain Sia*α*2,6Gal^15^. In contrast, avian influenza viruses preferentially bind sialic acids linked to galactose by an *α*2,3 linkage (Sia*α*2,3Gal). Therefore, a switch from Sia*α*2,3Gal to Sia*α*2,6Gal receptor-binding preference is considered an important step for avian influenza virus adaptation to mammals^16^. Nevertheless, enhanced Sia*α*2,6Gal binding is not entirely sufficient to make H9N2 viruses transmissible between mammals^12, 17, 18^. This suggests that other amino acid substitutions and/or an adequate constellation of internal genes is also required for efficient transmission of H9N2 viruses between mammals.

Due to the segmented nature of the influenza viruses, the RNA segments of two (or more) different influenza viruses can “reassort” when co-infecting the same host cell, thereby generating progeny viruses with properties different from the parental viruses^19^. In that sense, the sporadic isolation of avian H9N2 influenza viruses from pigs and humans in China increases the possibility of co-infections with endemic swine or human influenza viruses, which could result in the generation of novel reassortant viruses with enhanced transmissibility in mammals.

The 2009 pandemic H1N1 virus (pH1N1) is a swine-origin reassortant virus containing human, swine and avian influenza virus genes^20^. After its first appearance in 2009, the virus has spread around the world in both human and swine populations^21^. Moreover, novel reassortant viruses containing pH1N1 internal-protein and surface-protein genes from other endemic swine influenza virus have been isolated frequently from pigs worldwide^22–25^. In addition, H3N2 swine influenza viruses containing the pH1N1-derived matrix (M) protein gene (H3N2 variant) were isolated from 364 human cases in the US since 2011^26^. This suggests that pH1N1 internal-protein genes are compatible with several surface-protein gene combinations and allow for robust replication in mammals.

In ferret transmission studies, a reassortant H9N2 virus containing pH1N1 internal-protein genes readily transmitted to all contact animals by respiratory droplets^27^. In pigs similar reassortant viruses also showed increased transmission when compared to the parental H9N2. However, their transmission was still not as efficient as that of swine-adapted viruses like pH1N1^17, 28^. We previously demonstrated that serial passages of A/quail/Hong Kong/G1/1997 (H9N2) virus in pigs slightly enhanced replication and transmission in swine (Mancera Gracia *et al*.^29^). Although the resulting pig-adapted virus carried a number of mutations compared to the parental virus, transmission was still less efficient than that of naturally circulating viruses. In the present study, we have examined the replication and transmissibility after ten serial pig passages of a reassortant virus containing A/quail/Hong Kong/G1/1997 (H9N2) surface-protein genes and NP gene within the A/California/04/2009 (pH1N1) background to evaluate if the pH1N1 backbone confers an advantage in H9N2 adaptation to pigs. This serial passaging of the H9N2:pH1N1 reassortant resulted in a virus that replicated in and transmitted between pigs at high rates. The predominant mutation in the passaged reassortant virus was an aspartic acid to glycine at position 225 in the HA RBS. Therefore, our results showed that the combination of reassortment and mutations induced by the serial passages generated a virus with a predominant mutation at position 225 in HA RBS that replicated and transmitted at high rates in pigs.

## Results

### Serial passages of a reassortant H9N2 virus resulted in increased virus replication

**Figure 1:**
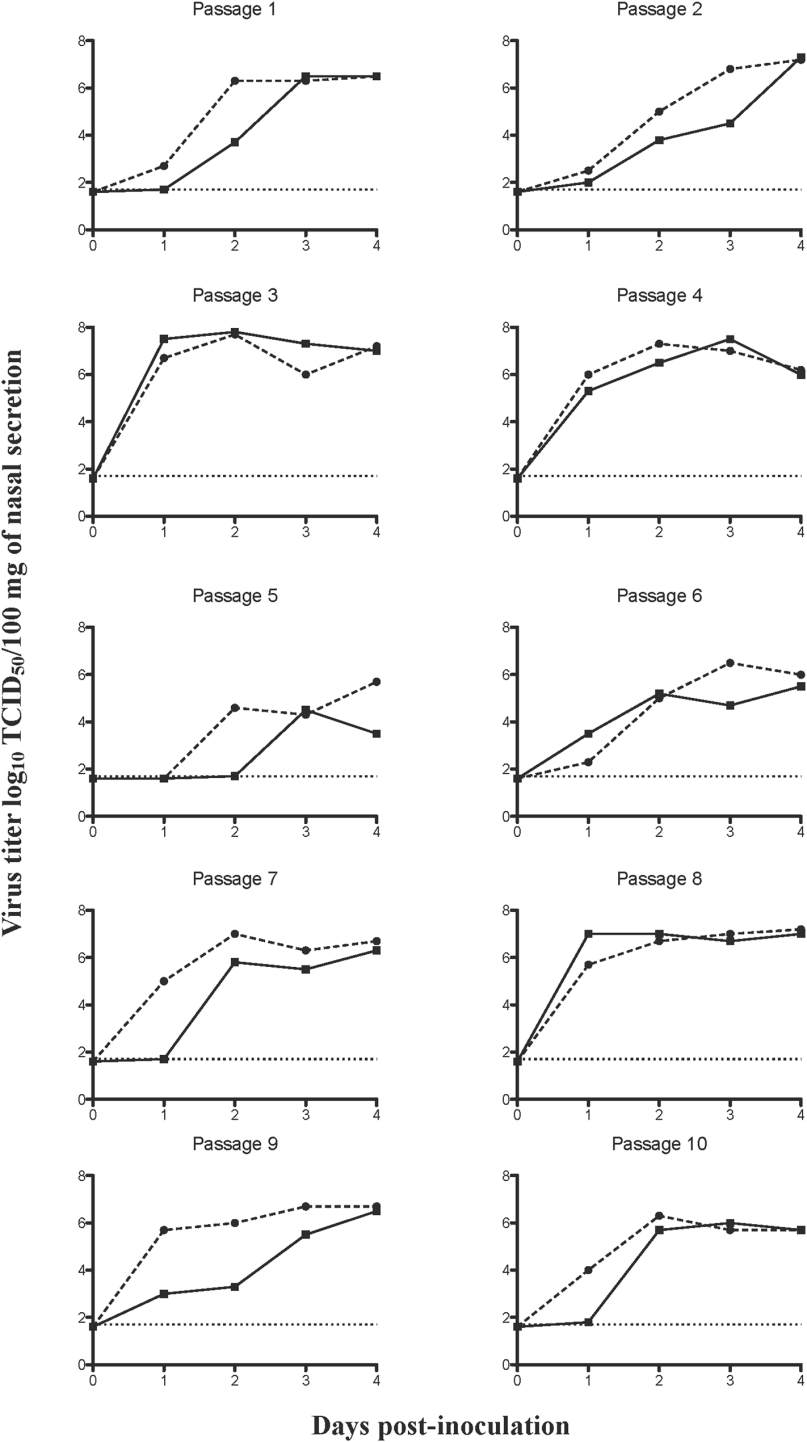
Nasal virus shedding of the reassortant H9N2 virus containing pH1N1 internal-protein genes (H9N2:pH1N1) during ten blind serial pig passages. Two pigs (solid line and dashed line) were used for in each passage, each line represents the virus titers in nasal swabs of an individual pig. The detection limit of the test is indicated with a dotted line at 1.7 log_10_TCID_50_/100 mg of secretion.

To evaluate if serial pig infections made reassortant H9N2 viruses better adapted to swine, ten blind serial passages were performed in influenza naïve pigs. For each passage two co-housed animals were intranasally inoculated with 10^6,5^ TCID_50_ of H9N2:pH1N1 (P0). To determine nasal virus excretion, individual pigs were swabbed daily from 0 to four days post-inoculation (dpi). None of the pigs showed clinical signs of disease. The reassortant H9N2 virus was detected in nasal swabs of all experimentally infected pigs. The highest amount of virus was excreted during passages three and eight (Fig. 1). At four days after infection, pigs were euthanized and samples from the entire respiratory tract were collected to determine the viral load. Infectious virus was recovered from the respiratory tract of all pigs during all ten serial passages (Table 1). Replication rates were higher in the upper respiratory tract (nasal mucosa: respiratory and olfactory parts) with all 40 out of 40 (100%) samples testing positive, when compared to the lower respiratory tract (trachea and lungs) with 81 out of 140 (58%) samples testing positive. In the first passage, no virus could be isolated from the lower respiratory tract. In contrast, during passages two, three and eight, the virus was detected in 17 out of 18 (93%) samples, indicating replication in the entire respiratory tract (Table 1).

**Table 1:** Distribution of reassortant H9N2 virus containing pH1N1 internal-protein genes in the respiratory tract during ten serial passages in pigs.

Number of passage	Pig number	Virus titer (log_10_ TCID_50_/gram of tissue) at day 4 post-inoculation^**a**^								
		Nasal mucosa respiratory part	Nasal mucosa olfactory part	Proximal trachea	Distal trachea	Apical + cardiac lobe right	Apical + cardiac lobe left	Diaphragmatic lobe right	Diaphragmatic lobe left	Accessory lobe
1	#1	5.3	5.5	<^b^	<	<	<	<	<	<
	#2	4.5	5	<	<	<	<	<	<	<
2	#3	5.7	5.7	2	4.2	<	3.3	2	3.5	2.3
	#4	7	5.7	4.3	4	4.3	2.5	2.5	3.8	2.5
3	#5	5.7	5.5	5.7	4.7	2.3	3.7	2.3	3	1.7
	#6	5.7	7.3	2.7	2	2.3	4.5	1.7	2.5	<
4	#7	5.5	6.3	2.3	3.8	1.7	<	3.7	<	1.7
	#8	6.3	5.5	5	4.7	5.7	4	4.5	4.5	2.7
5	#9	6	1.7	1.7	<	<	<	<	<	<
	#10	4.7	2.5	4.2	2.7	6	2,8	3.2	<	5.5
6	#11	5.5	3.5	1.7	<	<	<	<	<	<
	#12	6.3	4	<	<	<	<	<	<	<
7	#13	5.7	5.5	3.3	2.5	<	1.7	2.8	<	<
	#14	5.5	4.3	1.7	<	<	<	<	<	<
8	#15	6.3	7	4.3	5.5	5.3	5.5	5.5	5.5	4.5
	#16	6.7	6.3	2.5	2.8	<	3.2	4,8	3,3	4,3
9	#17	6.5	6.7	1.7	<	<	<	1.7	<	<
	#18	6	5.5	2.5	3.5	<	5.5	4.3	3.5	2,7
10	#19	7	5.3	1.7	<	2	<	3.5	2.5	<
	#20	6.8	3.7	1.7	5	5.5	2.2	<	4.7	<

^a^Virus titers are shown for each individual pig (#). ^b^<detection limit (1,7 log _10_ TCID_50_/gram of tissue).

### After seven serial passages in pigs, the reassortant H9N2:pH1N1 virus showed enhanced contact transmission

**Figure 2:**
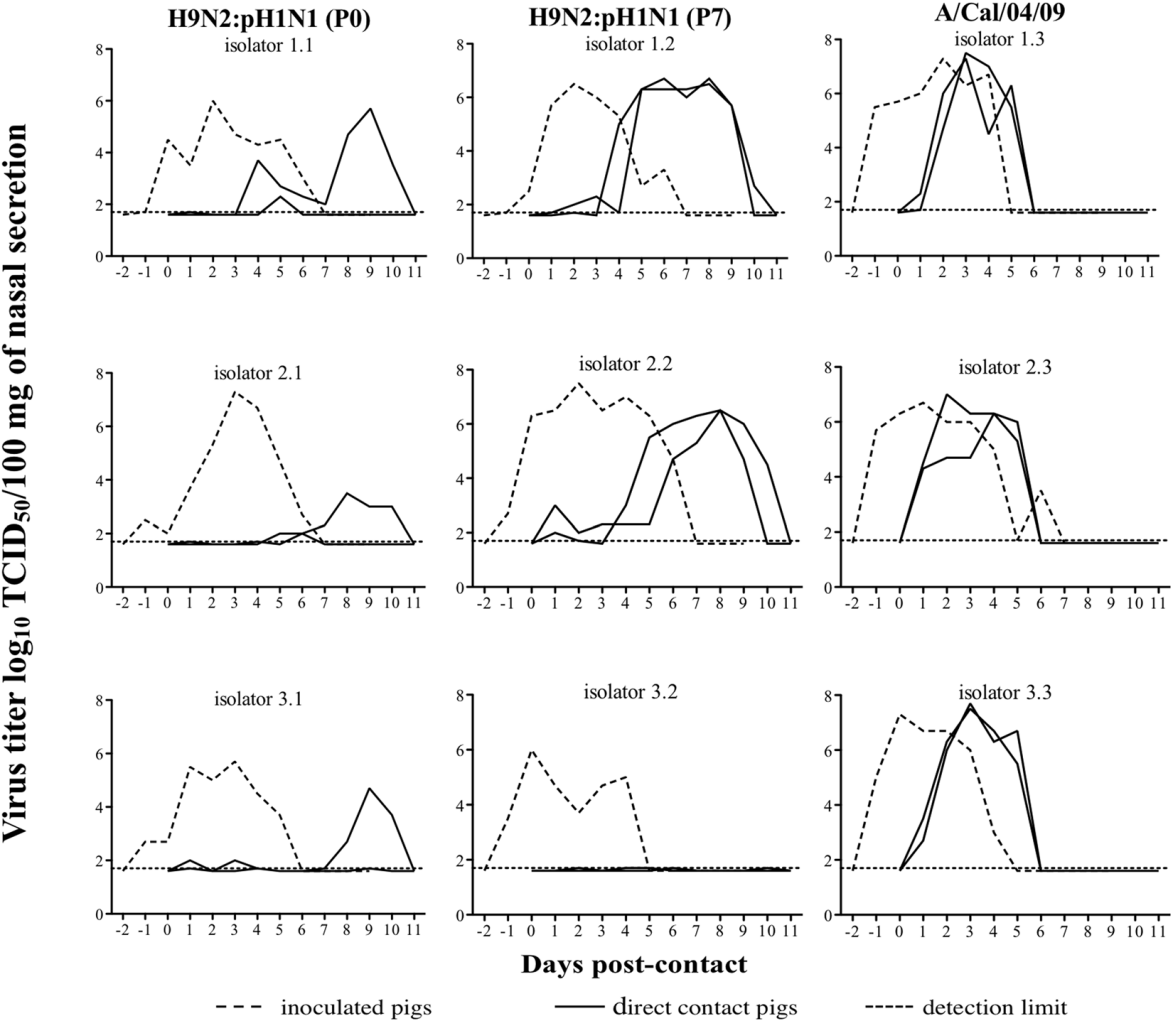
Nasal virus shedding and direct-contact transmission of H9N2:pH1N1(P0), H9N2:pH1N1(P7) and A/Cal/04/09 influenza viruses in pigs. Three pigs were intranasally inoculated with 6,5 log_10_ TCID_50_ of the indicated virus and housed in three separate isolators. Forty-eight hours later, two direct contact animals were co-housed with each inoculated pigs. Each graph is identified with a two digits number: the first one corresponds to the isolator number and the second to the virus tested. Therefore, each column represents a different virus and each row represents a different isolator. The detection limit (dotted line) of the test was 1.7 log_10 _TCID_50_/100 mg of secretion.

To compare the level of adaptation of the reassortant virus before and after serial passages in pigs, we performed direct-contact transmission experiments with the H9N2:pH1N1 (P0) virus and its descendent isolated after seven passages in pigs (H9N2:pH1N1 (P7)). The latter virus was chosen because virus titers in nasal swabs and the respiratory tract samples were highest at this passage, suggesting a substantial degree of swine-adaptation. As a positive control, we used a representative swine-adapted virus, the A/California/04/2009 (pH1N1) virus (A/Cal/04/09)^23^. All three viruses were amplified in MDCK cells before pig inoculation, and the transmission experiment was performed in triplicate for each virus. Figure 2 illustrates the nasal virus shedding of the inoculated and the co-housed direct-contact animals. All animals remained clinically healthy, based on clinical observation. All inoculated pigs excreted virus between days one and seven post-inoculation. A comparison of the averages of the areas under the curve (AUC) revealed a lower virus excretion for H9N2:pH1N1 (P0) (mean AUC 19.4) and H9N2:pH1N1 (P7) (24.0) than for A/Cal/04/09 (26.6), even though these differences were not statistically significant (p >0.05). All three viruses reached a maximum virus titer ≥6.5 log_10_ TCID_50_/100 mg of secrete. All inoculated animals developed neutralizing antibodies against the homologous virus and these were highest for the A/Cal/04/09 infected pigs (Table 2).

**Table 2:** Serum VN antibody titers in inoculated and direct contact pigs involved in transmission experiments measured.

Virus strain	Number of antibody positive pigs (range of antibody titers)							
	Inoculated pigs (n = 3)				Direct contact pigs (n = 6)			
	0	16	23	29 dpi^a^	0	14	21	28 dpc^b^
H9N2:pH1N1(P0)	0 (<2)	3 (16–128)	3 (32–256)	3 (32–192)	0 (<2)	3 (24–192)	6 (12–192)	6 (48–256)
H9N2:pH1N1(P7)	0 (<2)	3 (48–96)	3 (64–256)	3 (48–128)	0 (<2)	5 (2–256)	4 (96–256)	4 (64–192)
A/Cal/04/09	0 (<2)	3 (128–192)	3 (192–384)	3 (512–768)	0 (<2)	6 (192–1024)	6 (256–1536)	6 (192–1024)

^a^Dpi: days post-inoculation. ^b^Dpc: days post-contact.

As expected, all six A/Cal/04/09 direct-contact pigs excreted high amounts of virus (AUC >17) and seroconverted with VN titers ≥192. While all six direct-contact animals in the H9N2:pH1N1 (P0) group also seroconverted and excreted virus, only two shed virus with an AUC >6. In the H9N2:pH1N1 (P7) group, in contrast, just four out of six direct-contact animals excreted virus but all of them showed an AUC >21, similar to the A/Cal/04/09 group. The remaining two H9N2:pH1N1 (P7) direct-contact pigs did not excrete any virus and failed to seroconvert. VN titers in the six H9N2:pH1N1 (P0) and four out of six H9N2:pH1N1 (P7) direct-contact pigs were <256.

### Mutations associated with enhanced transmission

We next compared the variants present in the H9N2:pH1N1 (P0) virus population and the H9N2:pH1N1 (P7) virus isolated from the nasal mucosa of a directly inoculated pig and the direct-contact pig with the highest nasal excretion in the transmission study. By comparing the virus population from the directly-inoculated and the direct-contact pigs we aimed to determine if the mutations selected during the serial passaging were maintained after transmission or if the transmission itself favored the acquirement of a different set of mutations. In addition, the H9N2:pH1N1 (P0) virus stock was included to determine whether the detected variants in the passage seven virus were already present in the virus stock before pig adaptation. Illumina MiSeq deep sequencing was performed on full genome RT-PCR products of the samples. Sequencing of H9N2:pH1N1 (P0) virus revealed contamination of the H9N2:pH1N1 virus stock with H9N2 internal-protein genes, which were most pronounced for the PB1, NS and NP gene segments (see Table 3). Interestingly, passage of the reassortant H9N2:pH1N1 virus stock in pigs resulted in the selection of a virus that had lost these internal-protein-coding H9N2 gene segments, except for the NP segment which was still of H9N2-origin after seven pig passages. Sequence analysis of the H9N2:pH1N1 (P7) virus after contact transmission showed that the genetic makeup of this virus was nearly identical to the H9N2:pH1N1 (P7) stock virus. The selection for a H9N2 virus with the pH1N1-origin internal protein-coding gene segments suggests a strong genetic bottleneck that leads to elimination of reassortant viruses with H9N2 internal protein-coding gene segments upon viral passaging in pigs. The nonsynonymous variants that were detected at a frequency ≥5% in the H9N2:pH1N1 (P7) stock virus and the H9N2:pH1N1 (P7) virus isolated from the direct-contact pig after mapping the sequencing reads to the *de novo* assembled consensus sequence of the H9N2:pH1N1 (P7) stock virus are shown in Table 4. As a consequence of the contamination, the sequencing reads of the H9N2:pH1N1 (P0) stock virus were mapped to a reference genome composed of the *de novo* assembled consensus sequence of the H9N2:pH1N1 (P7) stock virus and the reference sequence for the A/quail/Hong Kong/G1/1997(H9N2) virus. The nonsynonymous variants detected at a frequency ≥0.5% in the H9N2:pH1N1 (P7) genome are shown in Supplementary Table S1.

**Table 3:** Mapping of the reads of the virus stock of H9N2:pH1N1 (P0), H9N2:pH1N1 (P7) and H9N2:pH1N1 (P7) isolated after direct-contact transmission to the reference genome sample obtained from A/quail/Hong Kong/G1/1997 (H9N2) and A/California/04/2009 (pH1N1).

Virus stock	Segment	Percentage of genes from … virus	
		A/quail/Hong Kong/G1/1997 (H9N2)	A/California/04/2009 (pH1N1)
H9N2:pH1N1 (P0)	PB2	6	94
	PB1	19	81
	PA	11	89
	HA	100	0
	NP	91	9
	NA	100	0
	M	26	74
	NS	91	9
H9N2:pH1N1 (P7)	PB2	0	100
	PB1	0	100
	PA	0	100
	HA	100	0
	NP	100	0
	NA	100	0
	M	0	100
	NS	0	100
H9N2:pH1N1 (P7) direct-contact	PB2	0	100
	PB1	2	98
	PA	0	100
	HA	100	0
	NP	100	0
	NA	100	0
	M	0	100
	NS	0	100

The viral gene segment is indicated along with the frequency (percentage) of sequence reads originating from either one of the parental strains.

**Table 4:** Variants present in the passage seven reassortant (H9N2:pH1N1(P7)) virus isolated from the nasal mucosa and from the nasal swab of the direct contact pig with the highest excretion.

Segment	Nucleotide position	Reference	Mutation	Amino acid change	Frequency nasal mucosa	Frequency nasal swab
PA	1260	T	A	Trp406Arg	—	5.09
	1990	TA	—	Leu649-	—	5.56
PB2	220	C	G	Thr58Ala	—	18.55
	1651	C	T	Thr535Met	46.74	—
	1891	T	C	Ile615Thr	8.09	—
HA	407	C	T	Leu119Phe	27.52	—
	750	A	G	Asp225Gly	47.43	99.70
NS	582	G	A	NS1:Gly179Gln NS2:Gly22Arg	30.45	—
	862	C	T	NS2: Ala115Val	—	25.83

The viral gene segment is indicated, along with the nucleotide position and substitutions, the predicted amino acid change and its position and the frequency (percentage) of sequence reads with the detected mutations. Only nonsynonymous substitutions with a frequency ≥5% in the reads are shown. The virus in the nasal mucosa sample was amplified on MDCK cells before sequencing.

The H9N2:pH1N1 (P7) stock virus showed variation above 5% at positions 535 and 615 of the basic polymerase 2 protein (PB2), position 119 of the HA and position 179 of the non-structural 1 protein (NS1) (Table 4). Nonetheless, those variants were not maintained after transmission to the contact pig. Interestingly, only one mutation associated with an amino acid substitution was selected after seven passages and maintained after transmission, *i.e*. the substitution of aspartic acid by glycine at position 225 of the HA RBS (HA-D225G). This mutation was absent (Supplementary Table S1) in the H9N2:pH1N1 (P0) virus stock and present in 47.4% and 99.7% of the sequences derived from the viral population isolated after seven passages and the transmitted virus, respectively. Two other mutations appeared *de novo* after transmission at a frequency >15%: a substitution of alanine to valine at position 115 of the NS1 and a substitution of threonine to alanine at position 58 of the PB2.

## Discussion

Since the late 1990s, avian H9N2 viruses have been isolated from pigs and humans, so far always as dead end events^3, 8, 30^. However, the continuous exposure of humans and pigs to avian H9N2 viruses might pose a real threat for public health if these viruses would acquire the genetic changes that allow them to transmit efficiently between mammals. In this study, we evaluated the efficiency of direct-contact transmission between pigs of a reassortant H9N2 virus containing pH1N1 internal-protein genes, and the effect of serial pig passages on transmission efficiency. We demonstrated, for the first time, that a pig-passaged reassortant H9N2:pH1N1 virus was excreted by more than half of the direct-contact pigs at titers similar to those of the parental pH1N1 virus.

Our data confirm the ability of the H9N2:pH1N1 reassortant viruses to infect pigs as was reported in previous studies^17, 18, 28^. In fact, virus excretion from the nasal cavity and replication in the respiratory tract were present at variable rates during the ten serial pig passages. This finding contrasts with a previous study from our group using the same parental H9N2 virus (A/quail/Hong Kong/G1/1997), however without reassortment with pH1N1 internal-protein genes (Mancera Gracia *et al*.^29^. In the latter study, replication and shedding of the parental H9N2 virus was slightly enhanced after four pig passages but it was completely lost after ten passages in pigs. This demonstrates that the presence of the well pig-adapted pH1N1-origin internal-protein genes may pose an advantage for the adaptation of H9N2 virus to pigs. While the H9N2:pH1N1 (P0) virus did not show replication in the lungs during passage one, it replicated homogeneously in the entire respiratory tract during passage two. Strikingly, the virus replication in the lungs then progressively decreased during passages four, five and six to increase again at passage seven. This suggests the presence of a selection bottleneck due to the genetic pressure generated by the experimental set up^31^. However, a deeper analysis of the viral population would be needed to elucidate the possible cause of the decrease in the replication rates.

We consider an influenza virus as swine-adapted when it succeeds to transmit between pigs with a similar efficiency as described for the endemic swine influenza viruses. Therefore, we compared pig-to-pig transmission of the seventh passage virus with that of the original reassortant and pH1N1 viruses. Whereas both reassortant viruses transmitted to direct-contact pigs, the seventh passage virus was clearly shed at higher titers by four out of six direct-contact pigs. This virus also showed higher transmission efficiency when compared with previous studies describing H9N2:pH1N1 reassortant viruses in pigs^17, 28^. In the aforementioned studies ≤50% of the direct-contact pigs excreted virus. This higher transmission rate suggests that the serial passaging in pigs contributed to the adaptation of the reassortant H9N2 virus. Yet of the six direct-contact pigs two animals that were housed in the same isolator did not excrete virus at all. This is most likely due to the low amount of virus excreted by the direct-inoculated pig (AUC 17.1), which not have reached a critical threshold to allow virus transmission. Further detailed genetic analyses of the virus excreted by the donor pig would be needed to better understand if there is, besides the low titer, also a genetic basis for the lack of transmission.

To elucidate the effects of the serial passaging and the pig-to-pig transmission on the virus population, we compared the viral diversity in the original reassortant virus with the virus isolated after seven passages in pigs and the virus excreted after direct-contact transmission. It should be mentioned here that we detected H9N2 internal-gene segments in our original reassortant virus stock. Interestingly, the serial passaging resulted in the purification of the original virus and, after seven passages only the NP-coding gene was completely of H9N2-origin. As in the original stock the H9N2-derive NP gene segment was present at 91%, this high percentage might have been the reason for the purification towards the H9N2-origin instead of towards the pH1N1-origin as seen for the other gene segments. However, the NS-coding gene showed the same percentage of contamination and was completely selected upon passaging. Although previous studies demonstrated that pH1N1-origin internal genes conferred an advantage in replication and transmission of influenza viruses in mammals^27, 32–36^, those studies focused either on the role of the PB2 and the M-coding genes or on the complete internal gene cassette. Not much is known about the exact role of pH1N1-origin NP-protein gene in the adaptation of influenza viruses to mammals. However, it has been described that the interaction between the NP and importin-α can influence the influenza virus host range. For example, the presence of the NP-N319K substitution was described as an important factor for the replication of H7N7 viruses in mice^37^. Nevertheless, both the H9N2 and the pH1N1 NP-protein genes lack that mutation. Thus, further analysis with reverse genetic generated reassortants would be necessary to elucidate if the combination of H9N2-origin NP-protein gene and pH1N1-origin internal genes has an effect in the replication and transmission of influenza viruses in mammals. Regarding the mutations detected during the analysis, only one of the mutations that appeared in the virus after seven passages and was maintained even after transmission, the HA-D225G substitution in the RBS. This mutation was therefore likely associated with the higher replication and transmission efficiency of the H9N2:pH1N1 (P7) virus. Interestingly, this mutation was present in all forty-seven swine and seventeen human H9N2 field isolates available in GenBank in November 2016. This mutation was also associated with enhanced replication and transmission of parental H9N2 after four passages in pigs (Mancera Gracia *et al*., *manuscript under review*). In the 1918 and 2009 pandemic H1N1 viruses this mutation has been associated with increased Siaα2,3Gal tropism^38, 39^. In pigs and humans Siaα2,3Gal receptors are mainly found in the lungs^13^. The presence of the HA-D225G mutation after seven passages may therefore explain the consistent replication in the lungs during passage eight. Although specific receptor-binding tests would be necessary to elucidate the exact role of the HA-D225G mutation within the HA-RBS, our data suggest that it may be a marker for adaptation of H9N2 viruses to swine.

In line with previous studies^17, 28^, this report demonstrated that avian H9N2 influenza viruses can reassort with pH1N1 internal genes, resulting in enhanced virus transmission in pigs when compared to the parental, non-reassorted H9N2 virus. The present study also underscores that repeated introduction of reassortant H9N2 viruses into the swine or humans may result in the selection of virus variants with a transmission efficiency close to that of the endemic swine or human influenza viruses. However, the lack of transmission detected in one of the contact groups used for transmission of passage seven virus, emphasizes the complexity of the adaptation process of an avian virus to a mammalian species and the need for additional research to better understand this crucial step in cross-species transmission of influenza viruses.

## Materials and Methods

### Viruses

The reassortant virus containing A/California/04/2009 (pH1N1) internal genes and A/quail/Hong Kong/G1/1997 (H9N2) HA and NA was produced by reverse genetics at the Department of Diagnostic/Pathobiology at Kansas State University. The virus was plaque purified, passaged three times in MDCK cells, characterized and experimentally validated^17^. Another passage in MDCK cells was made at the arrival of the virus to the Laboratory of Virology at Ghent University to generate a stock to be used in this study.

A/California/04/2009 (pH1N1) was kindly provided by the Centers for Disease Control and Prevention (US CDC). The virus underwent three passages in MDCK cells at the US CDC. Later one passage in eggs and one in MDCK cells were performed in the Laboratory of Virology at Ghent University before use.

### Pig passages and transmission studies

Forty-seven three-week-old piglets were purchased from a commercial herd free of swine influenza and porcine reproductive and respiratory syndrome virus. Upon arrival, pigs were confirmed seronegative to influenza virus with a commercial blocking anti-influenza A nucleocapsid ELISA (ID-VET) and by virus neutralization (VN) tests. Consecutive passages started with the housing of two pigs in a biosafety level-3 (BSL-3) HEPA filtered isolator, and their intranasal inoculation with three ml of phosphate-buffered saline (PBS) containing 10^6.5^ TCID_50_ of virus. Pigs were clinically monitored daily for general (depression, anorexia) and respiratory (coughing, dyspnoea, abdominal thumping, tachypnoea) symptoms. Nasal swabs were also collected daily from 0 to four dpi. At four dpi both pigs were euthanized with a lethal dose of pentobarbital sodium (≥100 mg/kg) in the jugular vein. The following tissue samples were collected for virus titrations: nasal mucosa respiratory part (i.e. nasal turbinates), nasal mucosa olfactory part (i.e. ethmoid labyrinth), trachea (distal and proximal half) and five different samples representative of the entire lung. The nasal mucosae (respiratory part) were pooled and a 20% (w/v) tissue homogenate was prepared and used to inoculate two pigs with this passage one virus inoculum. Ten blind (meaning that the viral load in the inoculum was not known prior to the infection) serial passages were performed in this way. Nasal swabs from all pigs, tissue samples and the inocula used for the subsequent passages were titrated on MDCK cells.

For each transmission experiment nine three-week-old pigs were used. At 0 dpi three pigs were housed in three separate BSL-3 HEPA filtered isolators (one index pig per isolator) and intranasally inoculated with 10^6.5^ TCID_50_ of the respective virus. Two dpi, two pigs were introduced in each isolator allowing direct contact with the inoculated pig. All animals were clinically monitored and nasal swabs for virus isolation were collected daily from all pigs during eleven days after cohousing. Sixteen dpi, the animals were relocated to a BSL-2 HEPA filtered isolation unit. Serum samples were collected at 0, 16, 23 and 30 dpi and 0, 14, 21 and 28 days post-contact.

### Ethical statement

All experimental procedures were conducted in accordance with the E. U. Animal Welfare Directives, and were authorized and supervised by the Local Ethical and Animal Welfare Committee of the Faculty of Veterinary Medicine, Ghent University (EC2013/63 number).

### Virus titrations

Nasal swabs from both nostrils were suspended in 1 ml PBS supplemented with 10% foetal bovine serum, 100 IU/ml penicillin and 100 μg/ml streptomycin and mixed vigorously at 4 °C for 1 hour. Tissue samples were weighed and grounded in PBS with 10 IU/ml penicillin and 10 µg/ml streptomycin to obtain 20% (w/v) tissue homogenates. Briefly, confluent monolayers of cells were inoculated with 10-fold serial dilutions of the sample. After five days of incubation at 37 °C with 5% CO_2_, virus positive MDCK cells were visualized by immunoperoxidase staining. The cells were fixed with 4% paraformaldehyde for ten minutes at room temperature and subsequently incubated with mouse anti-NP monoclonal HB-65 antibody (1:50, ATCC) for two hours. Incubation with horseradish peroxidase-conjugated goat anti-mouse polyclonal antibody (1:200, Dako) for one hour was followed by a development step with H_2_O_2_ as substrate and 3-amino-9-ethyl-carbazole (AEC) as precipitating agent. Virus titers were calculated by the method of Reed and Muench (1938) and expressed as log_10_ 50% tissue culture infective doses (TCID_50_) per 100 milligram (nasal swabs) or per gram (tissues).

### Virus neutralization assays

VN tests were performed on MDCK cells in 96-well plates, using 100 TCID_50_ of virus per well as previously described^40^. Briefly, 2-fold serum dilutions were incubated (1 h, 37 °C) with 100 TCID_50_ of MDCK cell-grown virus. MDCK cells (800.000 cells per ml) were incubated with the virus-serum mixture for 24 h, after which the virus positive cells were visualized by immunoperoxidase staining. Antibody titers were expressed as the reciprocal of the highest serum dilution that completely inhibited virus replication in MDCK cells.

### Data analysis

Nasal virus shedding of individual pigs was quantified by calculation of the AUC. The means of the AUCs of different viruses were compared using standard two–sample Mann-Whitney U tests. Differences were considered significant when p < 0.05. GraphPad Prism Software, version 5, was used for statistical analyses.

### RT-PCR

The RNA from the virus samples was isolated using the QIAamp Viral RNA Mini Kit (Qiagen, Valencia, CA). cDNA was synthesized with the Transcriptor First Strand Synthesis kit (Roche), already described^41^. Two separate reactions were performed, using primers specific for the influenza A vRNAs: CommonUni12G (GCCGGAGCTCTGCAGATATCAGCGAAAGCAGG) and CommonUni12A (GCCGGAGCTCTGCAGATATCAGCAAAAGCAGG). Subsequently, all eight genomic segments were amplified in one PCR reaction containing an 1:1 mix of CommonUni12G and CommonUni12A cDNA, primer CommonUni13 (GCCGGAGCTCTGCAGATATCAGTAGAAACAAGG) (200 nM) and the Phusion High Fidelity polymerase (Thermo Scientific)^41–43^. RT-PCR was performed as described^41^, with the first 5 PCR cycles performed with an annealing temperature of 45 °C (instead of 72 °C). PCR products were purified using the High Pure PCR Product Purification Kit (Roche) and elution of the DNA in sterile ultrapure water.

### Illumina MiSeq library preparation and sequencing

500 ng of each purified RT-PCR sample was sheared with an M220 focused-ultrasonicator (Covaris) set to obtain peak fragment lengths of 400 bp. Next the NEBNext Ultra DNA Library Preparation kit (New England Biolabs) was used to repair the ends and to add the Illumina MiSeq-compatible barcode adapters to 100 ng of fragmented DNA. The resulting fragments were size-selected at 300 to 400 bp using Agencourt AMPure XP bead sizing (Beckman Coulter). Then, indexes were added in a limited-cycle PCR (10 cycles), followed by purification on Agencourt AMpure XP beads. The fragments were analysed on a High Sensitivity DNA Chip on the Bioanalyzer (Agilent Technologies) before loading on the sequencing chip. After the 2 × 250 bp MiSeq paired-end sequencing run, the data were base-called and converted to Illumina FASTQ files (Phred +64 encoding).

### Next Generation Sequencing data analysis

Sequence data analyses were performed on the resulting Illumina FASTQ files (Phred 64+ encoding) using CLC Genomics Workbench (Version 7.0.3) following the analysis pipeline as described^41^. The processed sequencing reads were subsequently mapped to the *de novo* assembled consensus sequence of the H9N2:pH1N1 (P7) stock virus sequence. This consensus sequence is available through GenBank accession number (KY785904-KY785911). The processed sequencing reads for the H9N2:pH1N1 (P7) stock virus were mapped to a reference genome composed of the H9N2:pH1N1 (P7) stock virus sequence and the A/quail/Hong Kong/G1/1997(H9N2) virus genome. The raw sequencing data were submitted to the NCBI Sequence Read Archive where they can be found under project number: SRP095510.

## Electronic supplementary material

Supplementary Table S1: Variants present in H9N2:pH1N1(P0) virus stock.
