# Author Correction: A reassortant H9N2 influenza virus containing 2009 pandemic H1N1 internal-protein genes acquired enhanced pig-to-pig transmission after serial passages in swine

**DOI:** 10.1038/s41598-018-35446-9

**Published:** 2018-11-16

**Authors:** José Carlos Mancera Gracia, Silvie Van den Hoecke, Juergen A. Richt, Wenjun Ma, Xavier Saelens, Kristien Van Reeth

**Affiliations:** 1 Laboratory of Virology, Department of Virology, Parasitology and Immunology, Faculty of Veterinary Medicine, Ghent University, Merelbeke, Belgium; 2 Center for Medical Biotechnology, VIB, Ghent, Belgium; 3 Department of Biomedical Molecular Biology, Ghent University, Ghent, 9000 Belgium; 4 Department of Diagnostic Medicine/Pathobiology, College of Veterinary Medicine, Kansas State University, Manhattan, KS 66506 USA

Correction to: *Scientific Reports* 10.1038/s41598-017-01512-x, published online 02 May 2017

This Article contains an error in Reference 29:

‘Jose Carlos Mancera Gracia, Silvie Van den Hoecke, Xavier Saelens, Kristien Van Reeth & Mirco Schmolke. Effect of serial pig passages on the adaptation of an avian H9N2 influenza virus to swine. *PLoS One* **12**(4), e0175267 (2017)’

should read:

‘Mancera Gracia, J. C., Van den Hoecke, S., Saelens, X. & Van Reeth, K. Effect of serial pig passages on the adaptation of an avian H9N2 influenza virus to swine. *PLoS One* **12**(4), e0175267 (2017)’

