# Supplementary material for: A reassortant H9N2 influenza virus containing 2009 pandemic H1N1 internal-protein genes acquired enhanced pig-to-pig transmission after serial passages in swine
Source: Sci Rep. 2017 May 2;7:1323. doi: 10.1038/s41598-017-01512-x (PMC5430982; doi:10.1038/s41598-017-01512-x)
Supplement: Supplementary file 1 — Supplementary Table S1: Variants present in H9N2:pH1N1(P0) virus stock. [file 41598_2017_1512_MOESM1_ESM.pdf]

A reassortant H9N2 influenza virus containing 2009 pandemic H1N1 internal-protein genes acquired enhanced pig-to-pig transmission after serial passages in swine

José Carlos Mancera Gracia [1], Silvie Van den Hoecke [2,3], Juergen A. Richt [4], Wenjun Ma [4], Xavier Saelens [2,3], Kristien Van Reeth [1*]

[1]Laboratory of Virology, Department of Virology, Parasitology and Immunology, Faculty of Veterinary Medicine, Ghent University, Merelbeke, Belgium.

[2]Center for Medical Biotechnology, VIB, Ghent, Belgium.

[3]Department of Biomedical Molecular Biology, Ghent University, Ghent, 9000, Belgium.

[4] Department of Diagnostic Medicine/Pathobiology, College of Veterinary Medicine, Kansas State University, Manhattan, KS, 66506, USA.

*Corresponding author:

E-mail: kristien.vanreeth@ugent.be (KVR)

Supplementary Table S1: Variants present in H9N2:pH1N1(P0) virus stock. The sequencing reads of the H9N2:pH1N1 (P0) stock virus were mapped to a combined reference genome composed of the *de novo* assembled consensus sequence of the H9N2:pH1N1 (P7) stock virus and the reference sequence for the A/quail/Hong Kong/G1/1997(H9N2) virus. The nonsynonymous variants detected in the H9N2:pH1N1 (P7) genome above 0.5% are shown per genome segment.

PB2 (H1N1)

Reference Position	Reference	Allele	Frequency	Amino acid change
81	T	C	0.53	PB2:p.Ser12Pro
90	C	A	0.61	PB2:p.Arg15Ser
141	A	-	0.67	PB2:p.Lys32fs
145	A	G	1.16	PB2:p.Lys33Arg
175	C	A	0.69	PB2:p.Pro43His
196	T	A	0.87	PB2:p.Met50Lys
199	T	-	0.82	PB2:p.Met51fs
214	C	A	0.57	PB2:p.Pro56Gln
237	A	T	0.51	PB2:p.Met64Leu
242	C	A	0.80	PB2:p.Asp65Glu
242	C	G	0.55	PB2:p.Asp65Glu
244	T	C	0.55	PB2:p.Met66Thr
325	C	A	0.53	PB2:p.Pro93His
1651	C	T	100.00	PB2:p.Thr535Met
1933	G	A	11.11	PB2:p.Ser629Asn
1987	TACTGG	-	56.89	PB2:p.Ile647_Val649delinsIle
1993	T	-	3.36	PB2:p.Val649fs
2100	G	T	0.64	PB2:p.Gly685Trp
2101	G	T	0.62	PB2:p.Gly685Val
2138	G	T	0.51	PB2:p.Leu697Phe
2146	A	C	0.76	PB2:p.Glu700Ala
2157	-	AT	7.05	PB2:p.Tyr704fs
2232	G	T	0.54	PB2:p.Gly729Trp
2233	G	T	0.67	PB2:p.Gly729Val
2263	G	T	0.60	PB2:p.Arg739Leu

PB1 (H1N1)

Reference Position	Reference	Allele	Frequency	Amino acid change
74	AAAAATTCCAGCGCA	-	1.25	PB1:p.Leu10_Gln15delinsLeu
112	C	A	0.67	PB1:p.Pro23His
186	C	A	0.53	PB1:p.Gln48Lys
190	ACT	GAA	13.38	PB1:p.Tyr49_Ser50delins*Thr
192	T	-	4.14	PB1:p.Ser50fs
192	T	A	12.87	PB1:p.Ser50Thr
205	A	G	55.27	PB1:p.Lys54Arg
269	G	T	6.88	PB1:p.Glu75Asp
2146	C	A	0.60	PB1:p.Pro701His
2187	G	A	0.87	PB1:p.Val715Met
2224	G	T	0.63	PB1:p.Arg727Met

PA (H1N1)

Reference Position	Reference	Allele	Frequency	Amino acid change
117	G	T	0.52	PA:p.Gly25Trp
118	G	T	0.60	PA:p.Gly25Val
211	A	T	5.97	PA:p.Glu56Val
214	G	T	1.31	PA:p.Arg57Leu
247	C	A	1.23	PA:p.Pro68Gln
340	G	T	0.65	PA:p.Gly99Val
418	G	T	0.60	PA:p.Arg125Met
695	A	C	70.88	PA:p.Gln217His
938	G	T	1.82	PA:p.Glu298Asp

Supplementary Table S1: Variants present in H9N2:pH1N1(P0) stock virus (Continued).

PA(H1N1)

Reference Position	Reference	Allele	Frequency	Amino acid change
1260	T	A	2.67	PA:p.Trp406Arg
1696	G	T	0.54	PA:p.Arg551Met
1775	G	T	0.76	PA:p.Trp577Cys
1921	G	T	0.54	PA:p.Arg626Met
1922	G	T	0.68	PA:p.Arg626Ser
1944	G	T	0.56	PA:p.Gly634Trp
1957	G	A	1.28	PA:p.Arg638Lys
2062	G	T	0.68	PA:p.Arg673Met
2094	G	T	0.55	PA:p.Gly684Trp
2095	G	T	0.61	PA:p.Gly684Val
2095	GG	AA	0.69	PA:p.Gly684Glu
2097	G	T	0.55	PA:p.Gly685Trp
2107	A	G	0.82	PA:p.Glu688Gly
2140	G	T	0.59	PA:p.Trp699Leu

HA (H9N2)

Reference Position	Reference	Allele	Frequency	Amino acid change
180	C	A	0.56	HA:p.Pro43His
254	C	A	0.62	HA:p.Leu68Ile
300	C	A	0.76	HA:p.Pro83His
314	C	A	0.53	HA:p.Leu88Met
381	C	A	0.59	HA:p.Pro110His
383	G	T	0.57	HA:p.Gly111Trp
459	C	A	0.54	HA:p.Pro136Gln
575	C	A	0.58	HA:p.Gln175Lys
591	G	T	0.57	HA:p.Arg180Met
616	G	T	0.51	HA:p.Trp188Cys
630	C	A	0.67	HA:p.Pro193Gln
757	G	T	0.75	HA:p.Gln235His
821	G	T	0.74	HA:p.Gly257Trp
899	A	G	3.12	HA:p.Ser283Gly
996	C	A	0.60	HA:p.Pro315His
1158	G	T	0.63	HA:p.Gly369Val
1437	G	T	0.55	HA:p.Arg462Met
1589	G	T	0.68	HA:p.Gly513Trp
1590	G	T	0.57	HA:p.Gly513Val
1669	G	T	0.56	HA:p.Met539Ile

NP (H9N2)

Reference Position	Reference	Allele	Frequency	Amino acid change
321	G	T	0.62	NP:p.Gly86Trp
331	C	A	0.54	NP:p.Pro89Gln
376	G	T	0.62	NP:p.Trp104Leu
525	C	A	0.51	NP:p.Leu154Ile
547	C	A	0.54	NP:p.Pro161His
579	C	A	0.62	NP:p.Leu172Ile
583	C	A	0.83	NP:p.Pro173Gln
617	G	T	0.65	NP:p.Lys184Asn
624	G	T	0.54	NP:p.Gly187Trp
632	G	T	0.51	NP:p.Met189Ile
747	G	T	0.70	NP:p.Gly228Trp
810	G	T	0.56	NP:p.Gly249Trp
943	G	T	0.73	NP:p.Arg293Met
948	G	T	0.64	NP:p.Gly295Trp
1041	A	G	1.50	NP:p.Ser326Gly
1110	G	T	0.66	NP:p.Gly349Trp

Supplementary Table S1: Variants present in H9N2:pH1N1(P0) stock virus (Continued).

NP (H9N2)

Reference Position	Reference	Allele	Frequency	Amino acid change
1111	G	T	0.57	NP:p.Gly349Val
1355	G	T	0.62	NP:p.Lys430Asn
1356	G	T	0.53	NP:p.Gly431Trp
1442	G	T	0.51	NP:p.Gln459His
1443	G	T	0.73	NP:p.Gly460Trp
1447	G	T	0.72	NP:p.Arg461Leu

NA (H9N2)

Reference Position	Reference	Allele	Frequency	Amino acid change
167	C	A	0.69	NA:p.Pro43Gln
328	C	A	0.75	NA:p.Pro97Thr
329	C	A	0.61	NA:p.Pro97His
367	G	T	0.59	NA:p.Gly110Trp
368	G	T	0.57	NA:p.Gly110Val
377	G	T	0.90	NA:p.Trp113Leu
522	G	T	0.51	NA:p.Leu161Phe
540	G	T	0.69	NA:p.Leu167Phe
619	G	T	0.53	NA:p.Gly194Trp
620	G	T	0.71	NA:p.Gly194Val
811	G	T	0.62	NA:p.Gly258Trp
878	C	A	0.62	NA:p.Pro280His
1046	G	T	0.61	NA:p.Arg336Met
1066	G	T	0.54	NA:p.Gly343Trp
1084	G	T	0.62	NA:p.Gly349Trp
1119	G	T	0.56	NA:p.Met360Ile
1166	G	T	0.61	NA:p.Arg376Met
1318	G	T	0.59	NA:p.Gly427Trp
1415	G	T	0.56	NA:p.Gly459Val

M (H1N1)

Reference Position	Reference	Allele	Frequency	Amino acid change
92	C	A	0.69	M1:p.Pro16Gln
103	C	A	0.65	M1:p.Leu20Ile
301	G	T	0.52	M1:p.Gly86Trp
307	G	T	0.51	M1:p.Gly88Trp
308	G	T	0.51	M1:p.Gly88Val
314	C	A	0.57	M1:p.Pro90Gln
366	A	G	0.77	M1:p.Ile107Met
450	G	T	0.62	M1:p.Met135Ile
514	T	G	0.52	M1:p.Ser157Ala
670	A	G	0.66	M1:p.Thr209Ala
687	T	G	0.84	M1:p.His214Gln
703	G	T	0.52	M1:p.Gly220Trp
745	C	A	1.30	M1:p.Leu234Ile
762	C	T	1.29	M2:p.[Pro10Leu]
771	G	A	1.15	M2:p.[Ser13Asn]
774	A	G	1.20	M2:p.[Glu14Gly]<br>M1:p.[Met244Ile];
777	G	T	0.70	M2:p.[Trp15Leu]
780	A	G	1.12	M2:p.[Glu16Gly]
814	CA	TG	1.19	M2:p.Val27_Ile28delinsValVal
825	A	G	1.64	M2:p.Asn31Ser
833	G	T	0.53	M2:p.Gly34Trp
860	AC	CT	1.47	M2:p.Thr43Leu
951	G	T	0.64	M2:p.Arg73Met
963	AA	GG	1.02	M2:p.Gln77Arg

Supplementary Table S1: Variants present in H9N2:pH1N1(P0) stock virus (Continued).

M (H1N1)

Reference Position	Reference	Allele	Frequency	Amino acid change
978	G	A	1.13	M2:p.Ser82Asn

NS (H1N1)

Reference Position	Reference	Allele	Frequency	Amino acid change
61	CA	TG	3.90	NS1:p.[Thr5_Met6delinsThrVal]<br>NS2:p.[Thr5_Met6delinsThrVal]
98	A	G	2.75	NS1:p.Ile18Val
119	A	C	1.63	NS1:p.Asn25His
127	G	T	0.75	NS1:p.Leu27Phe
138	C	A	0.55	NS1:p.Pro31Gln
161	G	A	0.74	NS1:p.Asp39Asn
177	A	G	0.82	NS1:p.Lys44Arg
186	G	A	0.75	NS1:p.Gly47Asp
189	A	G	0.84	NS1:p.Asn48Ser
222	T	G	0.77	NS1:p.Leu59Arg
235	A	T	0.76	NS1:p.Gln63His
244	AT	GC	0.74	NS1:p.Glu66_Trp67delinsGluArg
272	A	G	0.58	NS1:p.Thr76Ala
329	C	A	0.75	NS1:p.Leu95Ile
342	C	A	0.66	NS1:p.Ser99*
355	C	A	0.73	NS1:p.Phe103Leu
364	G	T	0.51	NS1:p.Met106Ile
477	-	C	0.95	NS1:p.Leu144fs
502	G	T	2.33	NS1:p.Glu152Asp
505	G	T	0.52	NS1:p.Glu153Asp
507	G	A	0.56	NS1:p.Gly154Glu
557	T	G	1.07	NS1:p.[Tyr171Asp]
559	T	C	1.11	NS2:p.[Met14Thr]
578	G	A	1.51	NS1:p.[Val178Ile]
582	G	T	0.57	NS1:p.[Gly179Val];<br>NS2:p.[Gly22Trp]
612	G	A	1.30	NS1:p.[Gly189Asp];<br>NS2:p.[Val32Ile]
619	G	A	1.16	NS2:p.[Arg34Gln]
636	A	C	1.00	NS1:p.[Asn197Thr];<br>NS2:p.[Ile40Leu]
638	A	C	0.93	NS1:p.[Ile198Leu]
650	G	A	1.23	NS1:p.[Ala202Thr]
660	A	G	1.08	NS1:p.[Asn205Ser];<br>NS2:p.[Thr48Ala]
662	T	A	0.87	NS1:p.[Cys206Ser]
674	G	T	0.78	NS1:p.[Gly210Trp];<br>NS2:p.[Met52Ile]
680	C	T	1.48	NS1:p.[Pro212Ser]
683	T	C	1.40	NS1:p.[Ser213Pro]
688	A	C	1.04	NS2:p.[Tyr57Ser]
695	G	A	1.30	NS1:p.[Glu217Lys]
697	G	A	1.25	NS2:p.[Ser60Asn]
704	T	C	1.02	NS1:p.[*220Arg]
706	A	G	1.05	NS1:p.[*220Trp];<br>NS2:p.[Glu63Gly]
726	G	A	1.02	NS2:p.Gly70Arg
742	A	G	0.91	NS2:p.Glu75Gly
765	A	G	1.03	NS2:p.Met83Val
861	G	A	0.74	NS2:p.Ala115Thr
